# Supplementary material for: An open-source framework for large-scale, flexible evaluation of biomedical text mining systems
Source: J Biomed Discov Collab. 2008 Jan 29;3:1. doi: 10.1186/1747-5333-3-1 (PMC2276192; doi:10.1186/1747-5333-3-1)
Supplement: Additional file 1 — Evaluation platform source code. The additional file contains the software evaluation framework discussed in this paper. The most current release can be downloaded from SourceForge.net [1]. See the accompanying README file for instructions on installation and use. Also included in the distribution is a collection of UIMA wrappers for some commonly used BioNLP tools and annotated corpora. [file 1747-5333-3-1-S1.zip › bionlp_uima_repository-1.0.1/docs/api/src-html/edu/uchsc/ccp/uima/ae/nlp/stemmer/PorterStemmer_AE.html]

```
001    /*
002     *
003     * The Original Code is UCHSC UIMA.
004     *
005     * The Initial Developer of the Original Code is University of Colorado.  
006     * Copyright (C) 2005.  All Rights Reserved.
007     *
008     * UCHSC Utilities was developed by the Center for Computational Pharmacology
009     * (http://compbio.uchsc.edu) at the University of Colorado Health 
010     *  Sciences Center School of Medicine with support from the National 
011     *  Library of Medicine.  
012     *
013     *
014     * Contributor(s):
015     *   William A. Baumgartner <william.baumgartner@uchsc.edu> (Original Author)
016     *   
017     * Created: April 2005
018     * Dependencies:
019     * TODO: 
020     * Changes:
021     *      wab - Nov 2005 - altered to work with CCPAnnotation Type System
022     */
023    package edu.uchsc.ccp.uima.ae.nlp.stemmer;
024    
025    import java.util.Iterator;
026    
027    import org.apache.uima.analysis_engine.ResultSpecification;
028    import org.apache.uima.analysis_engine.annotator.AnnotatorConfigurationException;
029    import org.apache.uima.analysis_engine.annotator.AnnotatorContext;
030    import org.apache.uima.analysis_engine.annotator.AnnotatorInitializationException;
031    import org.apache.uima.analysis_engine.annotator.AnnotatorProcessException;
032    import org.apache.uima.analysis_engine.annotator.JTextAnnotator_ImplBase;
033    import org.apache.uima.cas.CASException;
034    import org.apache.uima.jcas.JCas;
035    
036    import edu.uchsc.ccp.uima.annotation.syntactic.CCPTokenAnnotation;
037    import edu.uchsc.ccp.uima.util.UIMA_Util;
038    import edu.uchsc.ccp.util.nlp.mention.SlotMentionTypes;
039    import edu.uchsc.ccp.util.nlp.stemmer.PorterStemmer_Util;
040    
041    /**
042     * This class incorporates the Porter stemming algorithm into a UIMA analysis engine framework.
043     * 
044     * @author William A. Baumgartner, Jr.
045     * 
046     */
047    public class PorterStemmer_AE extends JTextAnnotator_ImplBase {
048        private final boolean DEBUG = false;
049    
050        /*
051         * (non-Javadoc)
052         * 
053         * @see com.ibm.uima.analysis_engine.annotator.JTextAnnotator#process(com.ibm.uima.jcas.impl.JCas,
054         *      com.ibm.uima.analysis_engine.ResultSpecification)
055         */
056        // public void process(JCas aJCas, ResultSpecification arg1) throws
057        // AnnotatorProcessException {
058        //          
059        // // get document text
060        // String docText = aJCas.getDocumentText();
061        //          
062        // PorterStemmer ps = new PorterStemmer();
063        //          
064        // // get the token annotations from the JCas
065        // // for each annotation of type Token, get the word, stem it, get the
066        // result
067        //          
068        // Iterator annotationIter =
069        // aJCas.getJFSIndexRepository().getAnnotationIndex(Token.type).iterator();
070        // String token, stemmedWord;
071        // while (annotationIter.hasNext()) {
072        // Token annot = (Token)annotationIter.next();
073        // token = annot.getCoveredText();
074        //                          
075        // // stem word
076        // token = token.toLowerCase();
077        // ps.add(token.toCharArray(),token.toCharArray().length);
078        // ps.stem();
079        // stemmedWord = ps.toString();
080        //                          
081        // //System.out.println("STEM: " + token + " " + beginIndex + "-" + endIndex
082        // + " " + stemmedWord + " " + beginIndex + "-" +
083        // (beginIndex+stemmedWord.length()) );
084        // //System.out.println("STEMMER: " + token + " " + stemmedWord);
085        //                          
086        // annot.setStemmedText(stemmedWord);
087        //                          
088        //                          
089        //                          
090        // // // store stemmed word
091        // // StemmedToken annotation = new StemmedToken(aJCas);
092        // // annotation.setBegin(beginIndex);
093        // // annotation.setEnd(beginIndex + stemmedWord.length());
094        // // annotation.addToIndexes();
095        // // try
096        // // {
097        // // getContext().getLogger().log(Level.FINEST,"Stemmed: " + annotation);
098        // // }
099        // // catch(AnnotatorContextException e)
100        // // {
101        // // throw new AnnotatorProcessException(e);
102        // // }
103        // }
104        // }
105        private PorterStemmer_Util porterStemmer;
106    
107        /**
108         * For each TokenAnnotation, this method will determine the stem of the token, and fill in the Stem field for the TokenAnnotation.
109         * 
110         */
111        public void process(JCas jcas, ResultSpecification arg1) throws AnnotatorProcessException {
112            // cycle through all TokenAnnotations, adding lemma as you go
113    
114            Iterator annotationIter = jcas.getJFSIndexRepository().getAnnotationIndex(CCPTokenAnnotation.type).iterator();
115            while (annotationIter.hasNext()) {
116                    CCPTokenAnnotation annot = (CCPTokenAnnotation) annotationIter.next();
117                String tokenText = annot.getCoveredText();
118                tokenText = tokenText.toLowerCase();
119                porterStemmer.add(tokenText.toCharArray(), tokenText.toCharArray().length);
120                porterStemmer.stem();
121    
122                String stem = porterStemmer.toString();
123                debug("Setting stem for token: " + tokenText + " -- " + stem);
124                annot.setStem(stem);
125                try {
126                    UIMA_Util.addSlotValue(annot.getClassMention(), SlotMentionTypes.TOKEN_STEM, stem);
127                } catch (CASException e) {
128                    e.printStackTrace();
129                }
130            }
131    
132        }
133    
134        /**
135         * Initializes the PorterStemmerAnnotator by initializing a new PorterStemmer
136         */
137        public void initialize(AnnotatorContext ac) throws AnnotatorInitializationException, AnnotatorConfigurationException {
138            porterStemmer = new PorterStemmer_Util();
139            super.initialize(ac);
140        }
141    
142        private void debug(String message) {
143            if (DEBUG) {
144                System.err.println("DEBUG -- PorterStemmerAnnotator: " + message);
145            }
146        }
147    
148    }
```
